# Distinct DNA methylation patterns associated with treatment resistance in metastatic castration resistant prostate cancer

**DOI:** 10.1038/s41598-021-85812-3

**Published:** 2021-03-23

**Authors:** Madonna R. Peter, Misha Bilenky, Alastair Davies, Ruth Isserlin, Gary D. Bader, Neil E. Fleshner, Martin Hirst, Amina Zoubeidi, Bharati Bapat

**Affiliations:** 1 Lunenfeld-Tanenbaum Research Institute, Sinai Health System, 60 Murray Street, Toronto, ON M5T 3L9 Canada; 2 Department of Laboratory Medicine and Pathobiology, University of Toronto, Toronto, Canada; 3 Canada’s Michael Smith Genome Science Center, BC Cancer Agency, Vancouver, Canada; 4 Vancouver Prostate Centre, Vancouver, BC Canada; 5 Terrence Donnelly Centre for Cellular and Biomolecular Research, University of Toronto, Toronto, Canada; 6 Division of Urology, Departments of Surgery and Surgical Oncology, University Health Network, Toronto, Canada; 7 Department of Microbiology and Immunology and Michael Smith Laboratories, University of British Columbia, Vancouver, Canada

**Keywords:** Prostate cancer, Tumour biomarkers

## Abstract

Androgens are a major driver of prostate cancer (PCa) and continue to be a critical treatment target for advanced disease, which includes castration therapy and antiandrogens. However, resistance to these therapies leading to metastatic castration-resistant prostate cancer (mCRPC), and the emergence of treatment-induced neuroendocrine disease (tNEPC) remains an ongoing challenge. Instability of the DNA methylome is well established as a major hallmark of PCa development and progression. Therefore, investigating the dynamics of the methylation changes going from the castration sensitive to the tNEPC state would provide insights into novel mechanisms of resistance. Using an established xenograft model of CRPC, genome-wide methylation analysis was performed on cell lines representing various stages of PCa progression. We confirmed extensive methylation changes with the development of CRPC and tNEPC using this model. This included key genes and pathways associated with cellular differentiation and neurodevelopment. Combined analysis of methylation and gene expression changes further highlighted genes that could potentially serve as therapeutic targets. Furthermore, tNEPC-related methylation signals from this model were detectable in circulating cell free DNA (cfDNA) from mCRPC patients undergoing androgen-targeting therapies and were associated with a faster time to clinical progression. These potential biomarkers could help with identifying patients with aggressive disease.

## Introduction

Prostate Cancer (PCa) is an androgen driven disease, with androgen-deprivation therapy (ADT) remaining the most predominant treatment for metastatic PCa^1^. Although initially beneficial, resistance to ADT is inevitable, leading to metastatic castration resistant prostate cancer (mCRPC)^2,3^. These tumors often continue to rely on the androgen pathway despite castrate/low levels of androgens through various mechanisms, including mutations in the androgen receptor (*AR*) gene and extragonadal androgen production^4^. As a result, the treatment landscape for mCRPC includes therapies that target extragonadal androgen biosynthesis (abiraterone acetate) and AR activation (i.e. enzalutamide)^5–8^. Much focus in recent clinical trials is determining optimal therapy sequences at various stages of PCa, from the castration-naïve setting to the mCRPC state^9,10^. While all of these treatments have been shown to improve survival, resistance occurs through various AR driven mechanisms and alternative lineage re-programming, owing to the molecular heterogeneity of PCa, which could lead to treatment induced neuroendocrine prostate cancer (tNEPC)^11–13^. Tracking the molecular changes that occur with each treatment could facilitate personalized treatment decisions^14^.

Extensive genomic analysis of mCRPC tumors has revealed molecular aberrations beyond the *AR* gene, implicating several oncogenic pathways, such as cell cycle regulation, apoptosis, and DNA repair^11,15^. In addition, epigenomic changes that occur with the development of PCa and during treatment are also a major contributor to the phenotypic heterogeneity among patients^16^. These alterations include chromatin remodeling, transcriptomic changes modulated by non-coding RNA, and altered DNA methylation^17–19^. In mCRPC, DNA methylation patterns were better able to distinguish Adenocarcinoma-CRPC (CRPC-Adeno) from a highly aggressive form of the disease, Neuroendocrine-CRPC (CRPC-NE)^20^, which is characterized by low canonical AR activity, frequent loss of tumor suppressor genes (i.e. *RB1* and *TP53*), concomitant with expression of neuronal lineage markers (i.e. chromogranin A and neuron-specific enolase) as well as stem-cell like factors (i.e. CD44)^21^. While the molecular drivers of trans-differentiation from CRPC-Adeno to CRPC-NE are still emerging, epigenomic changes likely play a significant role^22^. For instance, DNA methyltransferase 1 (DNMT1) is highly expressed in CRPC-NE tumors compared to CRPC-Adeno^20,23^.

In order to investigate the epigenomic mechanisms that drive CRPC-NE development, we assessed methylation alterations during distinct stages of prostate cancer progression. Using cell lines derived from a xenograft model, we analyzed the DNA methylome during the transition from a hormone/castration-sensitive state to CRPC to enzalutamide-resistant (ENZR) CRPC, including both CRPC-Adeno and CRPC-NE phenotypes. In particular, we distinguished methylation patterns associated with the development of CRPC-Adeno from CRPC-NE, highlighting key mechanisms and potential therapeutic targets specific to these clinical states. In addition, we integrated our findings from this pre-clinical model with circulating cell free DNA (cfDNA) from mCRPC patients to identify potential biomarkers associated with aggressive disease.

## Results

### Changes in DNA methylation patterns associated with the development of CRPC and enzalutamide resistance

**Figure 1:**
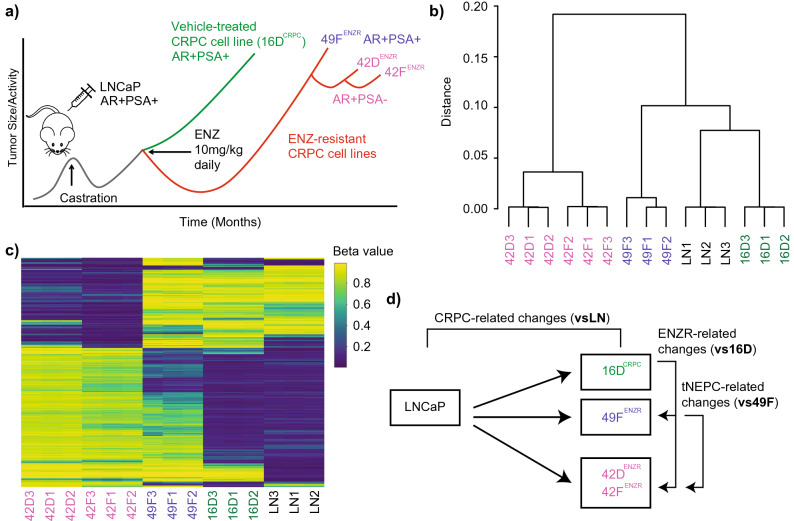
CRPC xenograft model and DNA methylation patterns across all cell lines. (**a**) Previously, LNCaP (LN) cells were injected into nude mice and allowed to grow into tumors. Following castration, CRPC-like cells/tumors emerge. The mice were either treated with vehicle-control or enzalutamide to create control CRPC cells (16D^CRPC^) or treatment resistant cells (49F^ENZR^/42D^ENZR^/42F^ENZR^), respectively. (**b**) Unsupervised hierarchical clustering of all detectable methylation array probes of each cell line (in triplicate) using the Ward linkage method. (**c**) Heatmap of the methylation levels (beta values) of the top 1000 differentially methylated probes (DMPs). (**d**) Commonly altered DMPs related to emergence of CRPC was found by comparing all CRPC cell lines to LN. Common enzalutamide resistance (ENZR) associated DMPs were determined through comparison with control CRPC ells (16D^CRPC^) and tNEPC related changes by comparing with 49F^ENZR^ cells.

Previously, CRPC and enzalutamide resistant (ENZR) CRPC cell lines were derived from a LNCaP xenograft mouse model (Fig. 1a)^24,25^. In this study, the methylome of the castration sensitive LNCaP cells (LN), vehicle control CRPC cells (16D^CRPC^) and three ENZR CRPC cells (49F^ENZR^/42D^ENZR^/42F^ENZR^) was assessed. Mimicking the clinical diversity of enzalutamide resistance, both AR-driven CRPC-Adeno (49F^ENZR^) and CRPC-NE/tNEPC (42D^ENZR^, 42F^ENZR^) phenotypes were examined. We performed methylation array profiling of > 485,000 probes/CpG sites across the genome. As expected, unsupervised clustering analysis of methylation levels across all probes show close clustering of replicates, with distinct methylation patterns among ENZR cells compared to LN and 16D^CRPC^ cells (Fig. 1b, c). The tNEPC cells, 42D^ENZR^ and 42F^ENZR^, demonstrated the most divergent methylation signals from LN cells.

### Increased differentially methylated probes/CpG sites with the development of ENZR and tNEPC

**Figure 2:**
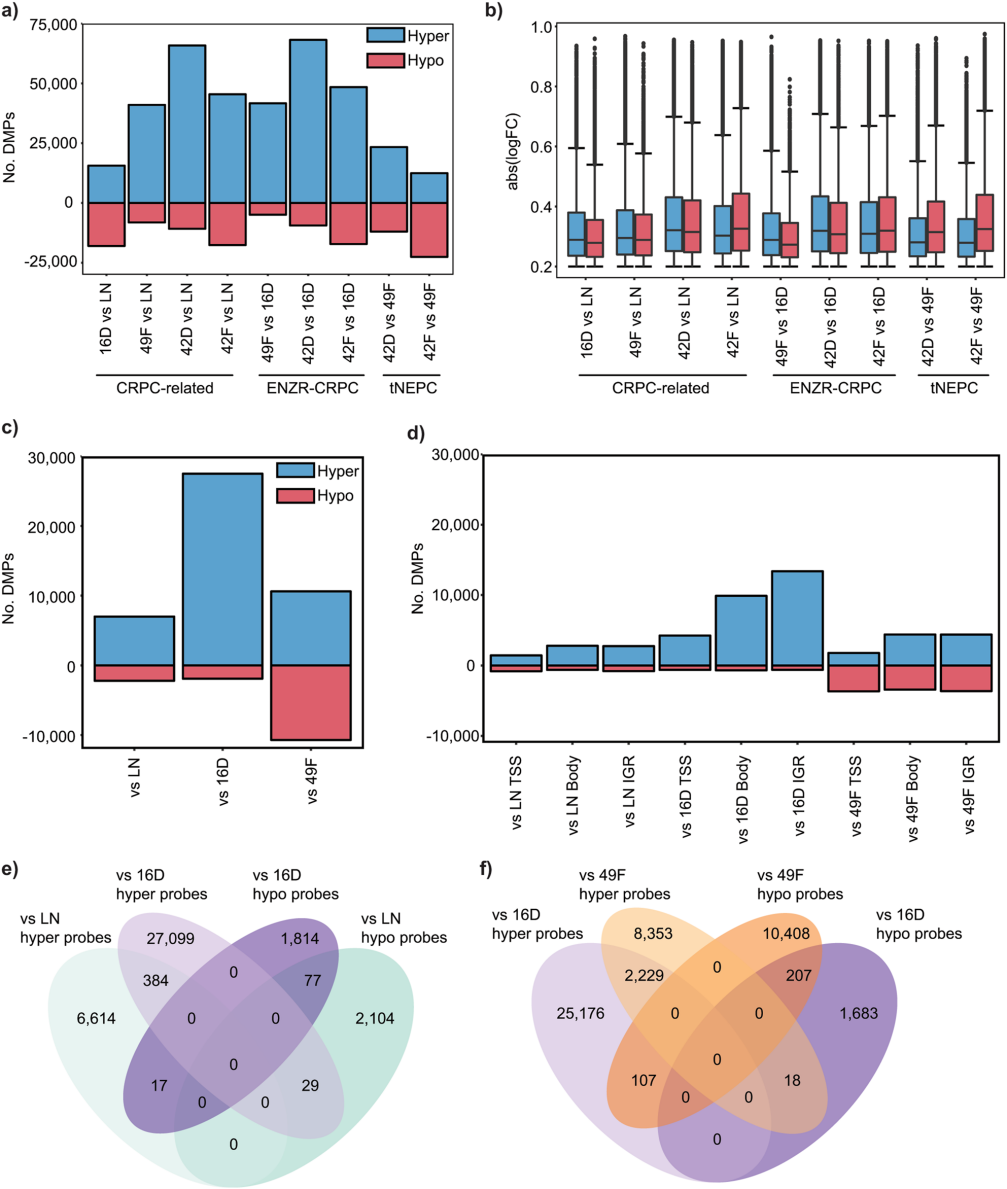
Overview of differentially methylated probes for all cell comparisons. (**a**) The total number of hypermethylated and hypomethylated probes for all comparisons is shown (**b**) Box plot summarizes the distribution of the absolute logFC values for all DMPs within each comparison. (**c**) Summary of the number of hypermethylated and hypomethylated DMPs shared by all vs LN, or vs 16D^CRPC^ or vs 49F^ENZR^ comparisons. (**d**) For TSS regions, gene bodies and intergenic regions, the total number of hypermethylated and hypomethylated DMPs is shown. (**e**) Venn diagram illustrates the extent of overlap between vs LN and vs 16D^CRPC^ DMPs according to methylation trend. (**f**) Similarly, the shared DMPs between vs 49F^ENZR^ and vs 16D^CRPC^ comparisons are illustrated.

We next performed differential methylation analysis to identify changes associated with: (a) the transition from castration sensitive to the castration resistant state (common changes between all CRPC/ENZR cells vs LN), (b) the development of enzalutamide resistance (all ENZR cells vs 16D^CRPC^) and finally (c) the tNEPC state (42D^ENZR^/42F^ENZR^ vs 49F^ENZR^) (Fig. 1d). We applied FDR (< 0.01) and logFC (≥ 0.2, fold-change) cut-offs to determine differentially methylated probes (DMPs) (Supplementary Tables 1–9). With the development of enzalutamide resistance (49F^ENZR^/42D^ENZR^/42F^ENZR^ vs LN), there was a larger number of DMPs compared to vehicle control cells (16D^CRPC^ vs LN), especially hypermethylated CpGs (Fig. 2a). Overall, all comparisons showed a similar logFC distribution, and were not significantly different between comparisons (Fig. 2b). The tNEPC cell lines, 42D^ENZR^/42F^ENZR^, tended to have the most DMPs (vs LN and vs 16D^CRPC^). The overall distribution of DMPs within transcriptional start sites/promoters (TSS, 0–1500 bp upstream), untranslated regions (UTRs), gene bodies and intergenic regions (IGR) did not vary across comparisons (Supplementary Figure 1a). Similar proportions of DMPs within CpG islands (CGIs), shores, shelves and open sea regions were also found across cell line comparisons (Supplementary Figure 1b). The proportion of hypermethylated versus hypomethylated DMPs for each genomic region confirmed tendency towards increased methylation of CpG sites analyzed in ENZR CRPC cells compared to parental (LN) or control CRPC (16D^CRPC^) cells (Supplementary Figure 1c–f).

To find critical methylation changes associated with the development of CRPC, we examined all cell lines vs LN comparisons, including 16D^CRPC^ vs LN and ENZR cells vs LN. This helped to refine key changes specific to overall development of CRPC. Similarly, common DMPs associated with enzalutamide resistance (49F^ENZR^/42D^ENZR^/42F^ENZR^ vs 16D^CRPC^), and the neuroendocrine phenotype (42D^ENZR^/42F^ENZR^ vs 49F^ENZR^) was also examined. With the development of CRPC and enzalutamide resistant states, the majority of common DMPs (vs LN or vs 16D^CRPC^) were hypermethylated (Fig. 2c). In contrast, there were nearly equal numbers of hypermethylated (10,600 DMPs) and hypomethylated (10,722 DMPs) DMPs commonly found between tNEPC cells compared to the CRPC-Adeno cells (42D^ENZR^/42F^ENZR^ vs 49F^ENZR^). Correlation analysis of common DMPs showed significant concordance of these shared DMPs across vs LN, vs 16D^CRPC^ and vs 49F^ENZR^ comparisons (Supplementary Figure 2). We further stratified these common DMPs by genomic location, with TSS/promoters, gene bodies and intergenic regions being the most represented (Fig. 2d).

We also examined whether there were any probes that overlapped going from the castration-sensitive to CRPC state and finally to the ENZR CRPC phenotype. There were 507 DMP sites associated with both CRPC development and enzalutamide resistance, the majority shared the same trend (i.e. 384 hypermethylated probes vs LN and vs 16D^CRPC^) and few changed directionality (i.e. 29 probes hypomethylated vs LN and hypermethylated in vs 16D^CRPC^ comparisons) (Fig. 2e). Interestingly, 107 probes were hypermethylated with the development of ENZR (vs 16D^CRPC^) but were hypomethylated in 42D^ENZR^/42F^ENZR^ cells compared to 49F^ENZR^ (Fig. 2f), suggesting that reduced methylation in 42D^ENZR^/42F^ENZR^ of these DMPs may be important for the transition to the tNEPC-like state. Indeed, global hypomethylation is associated with more genomic instability and found to be associated with PCa progression^26^. While 20/107 CpGs are located in intergenic regions, the remaining were found within/near several genes (Supplementary Table 10). We next examined the pathways and genes associated with ENZR and tNEPC development.

### Pathways and genes associated with CRPC development and enzalutamide resistance

**Figure 3:**
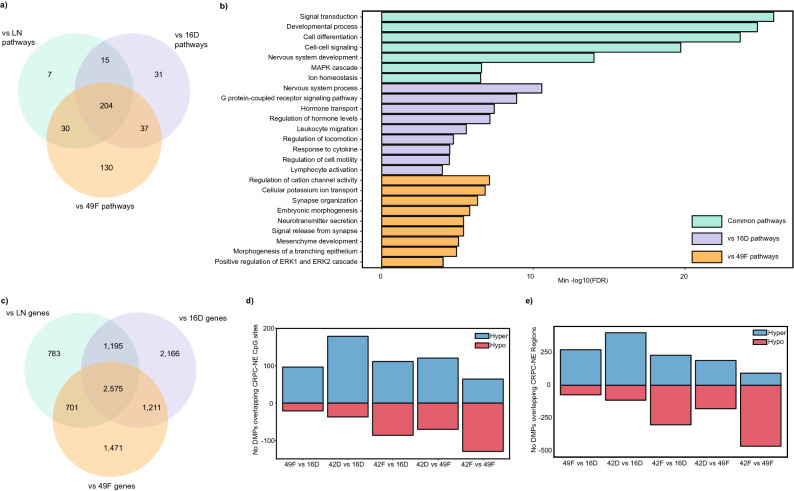
Summary of common pathways and comparison with tNEPC tumor-derived methylation patterns. (**a**) Venn diagram highlighting the common pathways associated with the development of CRPC, enzalutamide resistance and with the emergence of the tNEPC state. (**b**) Representative pathways with differentially methylated genes for all comparisons and ranked by the minimum -log10(FDR) values. (**c**) Venn diagram illustrates the overlap in the number of genes with DMPs between vs LN, vs 16D^CRPC^ and vs 49F^ENZR^ comparisons (TSS and gene bodies combined). (**d**) The DMPs from vs 16D^CRPC^ and vs 49F^ENZR^ comparisons were integrated with CRPC-NE vs CRPC-Adeno DMCs from a biopsy tissue study. The number of hypermethylated and hypomethylated DMPs that demonstrated the same methylation trends as CRPC-NE tumors is represented in the bar plot. (**e**) Similarly, DMPs were overlaid with known CRPC-NE regions previously detected in cfDNA and quantified for all ENZR cell lines.

We performed pathway analysis of all DMPs (Supplementary Tables 11–19) and examined pathways shared by all CRPC cells vs LN, ENZR cells vs 16D^CRPC^, and tNPEC cells vs 49F^ENZR^ comparisons. Interestingly, there were very few pathways with methylation changes specific to the development of CRPC (vs LN) alone, with the majority (204) shared across comparisons (Fig. 3a). Common pathways with the development of the CRPC state includes those involved in developmental processes, cellular differentiation, MAP kinase signaling and ion homeostasis (Fig. 3b). When we examined ENZR related pathways (49F^ENZR^/42D^ENZR^/42F^ENZR^ vs 16D^CRPC^), there were several hormone transport/secretion pathways implicated as well as immunomodulatory pathways, including leukocyte migration and lymphocyte activation. ENZR tNEPC cells compared to CRPC-Adeno cells tended to exhibit more embryonic, morphogenic and neuronal development pathways (Fig. 3b and Supplementary Tables 18–19). We next focused on genes associated with these DMPs, particularly near or within protein coding genes. Overall, we found 2575 genes with differentially methylated CpG sites (TSS/promoters and gene bodies combined) shared between all comparisons, and additional genes associated with the development of enzalutamide resistance and the tNEPC phenotype (Fig. 3c). However, as the array only samples a fraction of the ~ 28 million CpG sites in the genome, certain genes are better represented in terms of number of CpGs analyzed than others, with an average of 47% (range 30–60%) of genes with only 1–2 DMPs. In order to further refine important genes/pathways associated with disease progression, we integrated cell line methylation data with published data sets.

In a recent whole genome bisulfite sequencing study (WGBS) of mCRPC tumors, distinctive methylation patterns were observed amongst patients, including those patients with neuroendocrine disease^27^. We assessed the overlap of these methylated regions with DMPs from ENZR vs 16D^CRPC^ and tNEPC vs 49F^ENZR^ comparisons and further refined 209 genes with more than 5 DMPs. We calculated the net methylation change by scoring the ratio of hypermethylated to hypomethylated probes within each gene (Supplementary Figure 3). There were 81 genes that were hypermethylated with the development of ENZR and further hypermethylated among tNEPC cells. These genes included those involved in regulation of cell cycle (*CREB5*, *ERG*)^28,29^, tumor suppressors (*CTNNA2*, *OPCML*)^30,31^, as well as genes that regulate neurodevelopment (*AUTS2*, *SYNGAP1*)^32,33^. Furthermore, there were 19 genes that were hypermethylated in 49F^ENZR^ vs 16D^CRPC^, but hypomethylated in 42D^ENZR^/42F^ENZR^ cells, including the *AR* gene and development related genes (*PITX2*, *ROBO1/2*)^34,35^.

As several neurodevelopmental genes were implicated in our analysis, we wanted to examine whether these DMPs are also found within tNEPC tumor tissue. In a prior study analyzing CRPC-NE vs CRPC-Adeno biopsy tissue, differentially methylated CpG sites (DMCs) between these disease states were identified^20^. In a follow-up study, these CRPC-NE/tNEPC associated methylation patterns could be detected in matched cfDNA samples^36^. We first compared biopsy-derived DMCs with DMPs and found that tNEPC cells (42D^ENZR^/42F^ENZR^) tended to have more DMPs that overlapped with CRPC-NE tissue than 49F^ENZR^ (Fig. 3d). However, this biopsy study utilized reduced representation bisulfite sequencing (RRBS), which would have CpG sites not represented in the array in this study. We also examined whether vs 16D^CRPC^ and vs 49F^ENZR^ DMPs were detectable in cfDNA samples from confirmed CRPC-NE patients^36^, and found a similar pattern with more overlapping DMPs from the tNEPC cell lines (Fig. 3e). There were some CRPC-NE associated methylation patterns in 49F^ENZR^ cells, but not to the same extent as 42D^ENZR^/42F^ENZR^ cells.

### Promoter versus gene body methylation and impact on gene expression

DNA methylation alterations in promoters and gene bodies are known to impact gene expression^37^. For instance, hypermethylation in promoter/TSS regions can be associated with suppression of gene expression, whereas hypermethylation in gene body regions could be associated with increased expression. Among genes with DMPs in TSS regions (≥ 3 DMPs) and body regions (≥ 5 DMPs), the majority of genes were specifically altered in ENZR cell lines (Supplementary Figure 4a–b). In order to further investigate the impact of the DNA methylation changes on genes expression, especially with the development of tNEPC-like disease, we integrated DNA methylation data with RNA-seq expression data, which was previously generated for LNCaP, 16D^CRPC^, 42D^ENZR^ and 42F^ENZR^ cell lines^38^. We performed differential expression analysis comparing 16D^CRPC^ and ENZR cells to LN as well as 42D^ENZR^/42F^ENZR^ versus 16D^CRPC^, and identified genes that are upregulated or downregulated for these comparisons (Supplementary Tables 20–24). The overall number of differentially expressed genes tended to increase with the development of tNPEC phenotype (Supplementary Figure 4c), and very few overlapped across all vs LN comparisons, with most genes differentially expressed in 42D^ENZR^/42F^ENZR^ cells (Supplementary Figure 4d–g). We confirmed aberrant expression of AR regulated genes in all CRPC cell lines (16D^CRPC^/42D^ENZR^/42F^ENZR^ vs LN), including loss of TMPRSS2 expression (Supplementary Figure 5a)^39^. The tNEPC cell lines and not 16D^CRPC^ cells demonstrated reduced expression of prostate-specific antigen (PSA/*KLK3*) compared to LN (Supplementary Tables 20–22). The development of the tNEPC phenotype led to further alterations in known AR pathway regulated genes (42D^ENZR^/42F^ENZR^ vs 16D^CRPC^), including increased expression of *WNT5A*, which could be involved in the progression of aggressive disease^40,41^, and downregulation of a suppressor of metastasis, *NDRG1*^42^ (Supplementary Figure 5b).

For all vs LN differentially expressed genes, we assessed the following scenarios: (a) genes that are downregulated with promoter hypermethylation and no gene body hypermethylation, (b) genes that are upregulated with gene body hypermethylation and no promoter hypermethylation, (c) genes that were downregulated with gene body hypomethylation and no promoter hypomethylation, and (d) upregulated genes with promoter hypomethylation and no body hypomethylation (Supplementary Figure 6a–b). Compared to LN cells, there were only 12 genes with DMPs that were commonly downregulated in all CRPC cells (11 with scenario **a**, XKR6 with scenario **c**), including the ion transporter *SLC22A3*, which was shown to be downregulated in a subset of head and neck cancers^43^, and a regulator of neuronal development, *GPR126*^44^ (Supplementary Figure 6c). We next focused on genes specifically altered in tNEPC-like cell lines.

**Figure 4:**
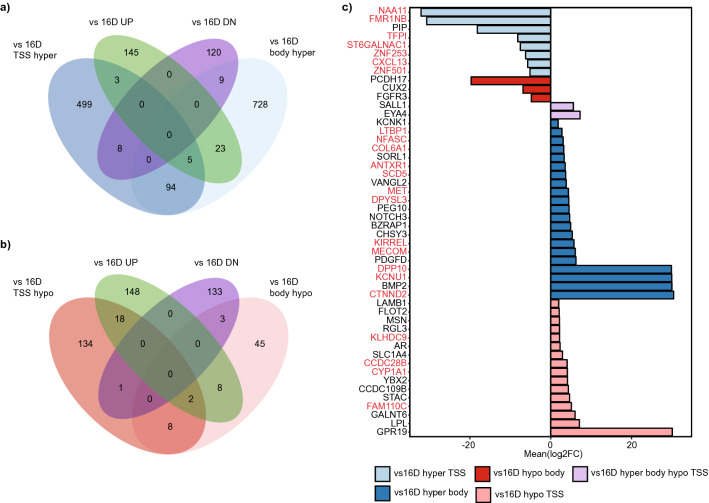
Integrated analysis of enzalutamide resistance related DNA methylation changes with gene expression changes. (**a**) Venn diagram examines genes that are hypermethylated in tNEPC cells vs 16D^CRPC^ TSS regions and/or gene bodies as well as with differential expression (UP = upregulated genes in 42D^ENZR^/42F^ENZR^, DN = downregulated genes). (**b**) Similarly, the extent of overlap between tNPEC cells vs 16D^CRPC^ hypomethylated genes is shown. (**c**) Bar plot shows all genes with differential expression and associated changes in DNA methylation in tNEPC cells. Mean log2FC for 42D^ENZR^/42F^ENZR^ vs 16D^CRPC^ is shown. Genes with similar methylation patterns in 49F^ENZR^ cells are indicated with pink text.

In terms of changes associated with development of neuroendocrine features (42D^ENZR^/42F^ENZR^ vs 16D^CRPC^), we examined the same scenarios as the LN comparisons (Fig. 4 a, b). All genes with DMPs in either TSS or body regions with accompanying expression changes are shown in Fig. 4c. While RNA expression data for 49F^ENZR^ cells was not available, we highlighted genes that showed similar methylation patterns in both tNPEC and CRPC-Adeno cells, which included promoters/markers of stem like phenotype (*ST6GALNAC1*, *ANTXR1*)^45,46^, and mediators of cell motility and growth (*CXCL13*, *COL6A1*)^47,48^. Alterations specific to the CRPC-NE phenotype, included mediators of development (*BMP2*, *NOTCH3*)^49,50^, and regulators of epithelial–mesenchymal transition (*FLOT2*, *PCDH17*)^51,52^. Interestingly, 42D^ENZR^/42F^ENZR^ cells also exhibited increased expression of *PEG10*, which was shown to be upregulated in CRPC-NE tumors^53^. In addition, tNEPC cells showed a slight increase in AR expression with hypomethylated TSS DMPs, suggesting that the AR pathway may still be utilized, but in a non-canonical fashion as these cells do not express PSA^38^. Few of these genes with combined methylation and gene expression changes are potentially regulated by the AR, including *CTNND2*, *CUX2* and *CXCL13*^39^. In particular, increased expression of a Wnt pathway gene, *CTNND2*, could be involved in resistance to AR targeting therapies^54^.

### ENZR-related methylation patterns in circulating DNA from mCRPC patients

**Figure 5:**
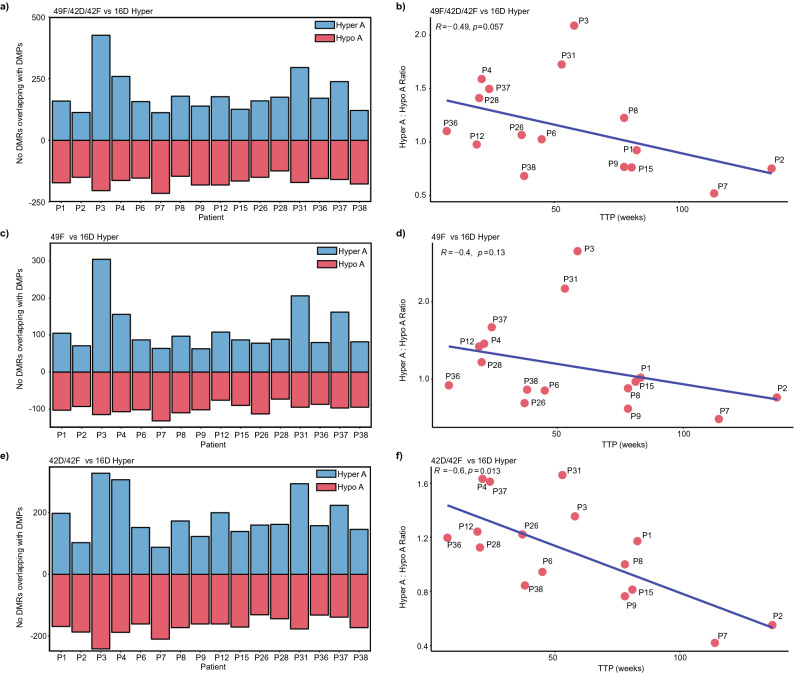
CRPC-NE related DMPs as potential biomarkers associated with clinical progression. The cell line DMPs that overlapped with cfDNA DMRs obtained from comparing the pre-treatment visit (visit A) and around 12-weeks during treatment (Visit B) were assessed. (**a**) The number of A vs B DMRs that overlapped with hypermethylated DMPs from comparing 49F^ENZR^/42D^ENZR^/42F^ENZR^ vs 16D^CRPC^ was quantified and separated by methylation trend for each patient (hypermethylated or hypomethylated in visit A vs B). (**b**) The ratio of these cfDNA DMRs (hypermethylated to hypomethylated) was correlated with TTP (Spearman rho and p value is shown). (**c**) Similarly, the number of DMRs that overlapped with 49F^ENZR^ vs 16D^CRPC^ hypermethylated DMPs (not found in 42D^ENZR^/42F^ENZR^) is shown in the barplot, and (**d**) correlation analysis with TTP is shown. (**e**) Finally, the number of cfDNA DMRs that contained hypermethylated DMPs from 42D^ENZR^/42F^ENZR^ vs 16D^CRPC^ (not 49F^ENZR^) was calculated, and (**f**) the ratio was also correlated with TTP.

Recently, we conducted a study collecting sequential cfDNA samples from mCRPC patients receiving androgen-targeting treatments, including enzalutamide and abiraterone acetate^55^. We tracked cfDNA methylation changes using genome-wide sequencing for each patient starting from prior to initiation of treatment (Visit A), at around 12 weeks during treatment (Visit B), and upon clinical progression (Visit C). When we examined the overlap between differentially methylated regions (DMRs) in cfDNA with CRPC-NE tissue related DMCs^20^, we found that patients that harbored a higher ratio of hypermethylated cfDNA DMRs to hypomethylated cfDNA DMRs (Visit A vs B) tended to demonstrate a faster time to clinical progression (TTP)^55^. Similarly, we examined the ENZR cell-line DMPs that were altered between: (1) 49F^ENZR^/42D^ENZR^/42F^ENZR^ and 16D^CRPC^ cells, (2) 49F^ENZR^ and 16D^CRPC^ alone, and (3) 42D^ENZR^/42F^ENZR^ and 16D^CRPC^ cells alone. Firstly, DMPs that were hypermethylated in all ENZR cell lines (49F^ENZR^/42D^ENZR^/42F^ENZR^) were overlaid with cfDNA DMRs (Visits A vs B). The ratio of these hypermethylated to hypomethylated DMRs (A vs B) was then correlated with TTP. There was a trend towards faster TTP for patients with increased cfDNA methylation at visit A for these regions (P = 0.057) (Fig. 5a, b). This trend was not observed for hypermethylated DMPs only found in CRPC-Adeno/49F^ENZR^ cells (Figs. 5c, d). However, when we overlapped cfDNA DMRs with hypermethylated DMPs from the tNEPC cell lines 42D^ENZR^/42F^ENZR^, there was a significant correlation with faster TTP (Figs. 5e, f). In order to assess whether this overlap between the array and cfDNA sequencing datasets was due to chance, we performed random sampling of the array dataset and compared the overlap of these probes with the cfDNA DMRs for each patient (Supplementary Table 25). There was a significant difference between the tNEPC-derived DMPs and randomly selected probes, suggesting that the tNEPC cell line derived methylation signals found within cfDNA samples is likely not due to random chance.

No correlation was observed for hypomethylated DMPs from any of these cell line comparisons (Supplementary Figure 7a–f). There was a slight correlation trend for DMPs hypermethylated in 42D^ENZR^/42F^ENZR^ vs 49F^ENZR^ (Supplementary Figure 7 g), but was not significant for hypomethylated DMPs (Supplementary Figure 7 h). These findings suggest that tNEPC related methylation signals could serve as potential biomarkers. Some patients with faster TTP continued to exhibit high PSA levels in circulation^55^, which highlights disease heterogeneity, as these patients could harbor both CRPC-Adeno and CRPC-NE tumor cells.

## Discussion

Overall, our analysis of the DNA methylome from the castration naïve state to treatment resistant CRPC suggests complex epigenetic changes in advanced PCa. We were able to track the changes in the methylome after successive treatments, including initial castration (representing conventional ADT) leading to CRPC state/cells followed by acquired resistance following enzalutamide treatment. Thus, mimicking progressive methylome changes as resistance emerges to various rounds of androgen-targeting treatment. In current clinical practice, the landscape of therapy sequences is complex, but still involving several types of androgen-targeting agents, chemotherapy, and for patients harboring CRPC-NE, potentially platinum-based therapies^21^. With each therapy, there are molecular alterations (genomic and epigenomic) that need to be tracked to determine optimal therapy sequences^11,27^. Therefore, it is important to distinguish the molecular alterations associated with androgen-pathway dependent disease, which could still be sensitive to androgen targeting agents and aberrations associated with tNEPC, which may benefit from other treatments. Furthermore, these molecular distinctions could help identify novel therapies for aggressive PCa. While this study was limited to a few representative cell lines, the advantage of this pre-clinical model was the ability to track the methylome at various stages of progression. Indeed, we observed methylation changes similar to those found in tumor tissue from tNEPC patients^20^.

In this study, we highlighted key methylome changes associated with the initial development of CRPC, then those following enzalutamide resistance and finally emergence of tNPEC-like disease. Despite sampling only a small fraction of the CpG sites in the genome, methylation alterations occurred in all genomic regions (i.e. promoters, gene bodies, and CGIs), and mostly within ENZR cells, especially in CRPC-NE/tNEPC like cells. While precise CpG sites may not have overlapped across all comparisons, several common genes and pathways were implicated within all ENZR cells, including those involved in developmental processes and hormone regulation, with more neurodevelopmental and morphogenic pathways enriched in tNEPC cells.

Interestingly, changes in the methylome appeared to have a potential impact on gene expression. While RNA data for CRPC-Adeno cells was not available, we noted similar methylation patterns in all ENZR cells, which was mostly associated with gene repression, including *SLC25A43*, which is deleted in certain breast tumors^56^, and a potential repressor of hypoxic response, *ZFP36L1*^57^. Common ENZR upregulated genes involved in metastasis, *COL6A1*^48^ and *ANTXR1*^46^, were also demonstrated. The genes implicated in all ENZR cells suggest potential targets beyond AR directed therapeutics that could impact growth and metastasis of both CRPC-Adeno and CRPC -NE cells, which requires further investigation. In addition, there were expression changes specific to tNEPC cells, including a known upregulated gene in CRPC-NE lesions, *PEG10*, which can regulate cell growth and invasion^58^. Other promoters of invasion/metastasis were also upregulated in tNEPC cells, such as *FLOT2*^51^, *LAMB1*^59^, and *GPR19*^60^. Although there was a slight increase in AR expression in ENZR/tNEPC cells, a previous study demonstrated siRNA knockdown of AR expression in these cells did not interfere with proliferation^38^. Furthermore, there are subsets of tumors with neuroendocrine features that also express AR, suggesting extensive plasticity in this disease state^13,61^. There were gene expression changes associated with low/altered AR activity, including reduced expression of TMPRSS2 and PSA/KLK3; however, many of these genes did not exhibit substantial methylation changes. This could be due to under-representation of CpGs near/within these genes in the array. Moreover, we observed that methylation changes in diverse pathways contributing to epigenomic instability in the tNEPC state.

We also assessed the potential of these methylation patterns as biomarkers of treatment resistance. By integrating the cell line methylation patterns with previously generated methylation profiles of cfDNA from mCRPC patients, we found potential markers of NE-like disease before patients initiated androgen-targeting agents. These markers were specific to the tNPEC cell lines and not CRPC-Adeno cells. In our prior study, extent of overlap between cfDNA DMRs and CRPC-NE tissue-derived signals also demonstrated a similar tendency towards faster clinical progression^55^. These combined findings highlight potential predictive biomarkers of resistance to androgen-targeting agents, requiring further validation in additional cohorts of patients.

## Methods

### CRPC cell line model

The CRPC cell line model was previously developed though serial transplantation of LNCaP-CRPC xenografts followed by castration and under the pressure of enzalutamide (10 mg/kg/d) or vehicle control^24,25,38^. This model was generated in accordance with approved animal use protocols in the Vancouver Prostate Centre, as specified by the Declaration of Helsinki for animal research. Cells were purified from xenografted tumors representing various stages of PCa progression: pre-castration LNCaP tumor cells (LN), vehicle treated CRPC (V16D^CRPC^) and ENZR cells (42D^ENZR^, 42F^ENZR^ and 49F^ENZR^). While all ENZR cell lines express AR, 49F^ENZR^ continues to secrete PSA and acquired an *AR* activating mutation, whereas 42D^ENZR^ and 42F^ENZR^ cells are PSA negative and do not possess this mutation^38^. Furthermore, 42D^ENZR^/42F^ENZR^ express neuroendocrine lineage markers (i.e. chromagranin A and neural cell adhesion marker 1) as well as stem-like properties^38^.

### DNA methylation and pathway analysis

DNA from each cell line was extracted using the QIAamp DNA mini Kit (Qiagen, Hilden, Germany) and quantified using Qubit dsDNA High Sensitivity Assay Kit (ThermoFisher Scientific, Waltham, MA, USA). We performed bisulfite conversion of 500 ng of DNA for each sample (in triplicate) using the Zymo EZ DNA Methylation kit (Zymo, Irvine, CA, USA). This was followed by methylation analysis using the Infinium HumanMethylation450 BeadChip array using manufacturer’s protocol (Illumina, San Diego, CA, USA). For each CpG site, beta value was calculated [β = Methylated allele intensity (M)/(Unmethylated allele intensity (U) + Methylated allele intensity (M) + 100)]. The Chip Analysis Methylation Pipeline (ChAMP) was used to analyze this dataset by first filtering out probes with low detection, SNP-related probes, and cross-reactive/multi-hit probes^62^. To adjust for probe type bias, normalization was performed using the BMIQ method^63^. Differentially methylated probes (DMPs) were then identified comparing each cell line with FDR < 0.01 and delta beta/log fold change (logFC) ≥ 0.2. All DMPs were annotated according to genomic location (i.e. TSS sites, genes). The champ.GSEA function was utilized to perform Gene Set Enrichment Analysis/Pathway analysis. Since the number of CpGs per gene varies, the gometh function was implemented to correct for this bias^64^.

### Differential expression analysis

RNA expression data was previously published for LNCaP, 16D^CRPC^, 42D^ENZR^ and 42F^ENZR^ cell lines^38^. Alignments were performed using the STAR aligner and the hg19 genome build was used^65^. RPKM assignment and quantifications was done with CEMT RNA-seq pipeline using the Ensembl v75 gene models (http://www.epigenomes.ca/data/CEMT/methods/RNA-Seq.html). Differential expression analysis was performed using the DEfine algorithm^66^. Chi-squared *P*-value was estimated for every gene and Benjamini–Hochberg false discovery rate was applied (FDR = 0.05).

### Additional statistical analysis

Unsupervised hierarchical clustering analysis of overall beta values was performed using the Ward clustering method in the base package of R (v3.5.3). Data was visualized using the VennDiagram package, the ggplots2 package, and heatmaps generated using the ComplexHeatmap package. Comparison with cfDNA-derived differentially methylated regions (DMRs) was performed using a published dataset^55^. Briefly, cfDNA was collected from patients receiving enzalutamide or abiraterone acetate treatment at various timepoints, including baseline/prior to treatment initiation visit (A), around 12-weeks during treatment (visit B), and upon clinical progression (visit C). Genome-wide methylation sequencing analysis was performed followed by differential methylation analysis between study visits. Intra-patient DMRs were identified using the DMRHunter tool for each patient by comparing all visits^55^. The extent of overlap between cfDNA DMRs with ChAMP-derived DMPs from various cell line comparisons was calculated for each patient. The ratio of hypermethylated to hypomethylated DMRs (visit A vs B) was estimated and Spearman correlation analysis with time to clinical progression was assessed. To determine whether this overlap between datasets was due to random chance, we calculated the proportion of DMRs that overlapped with tNEPC DMPs as well as the percentage of these tNEPC DMPs that overlapped with the 450 K array probes for each patient (X%). We then randomly sampled (X%) probes 10,000 × and determined the mean number of probes that overlapped with patient DMRs. We compared the proportion of random probes vs tNEPC probes within cfDNA DMRs using the one-proportion z-test.

All patients in this study provided informed written consent in accordance with approved institutional Research Ethics Board protocols from University Health Network (UHN) and Sinai Health System (SHS). Patients consented to the publication of study findings with unique study identifiers, which are not linked to personal health information. All work in this study was performed in accordance with the Declaration of Helsinki.

## Supplementary Information

Supplementary Information 1.

Supplementary Information 2.

Supplementary Information 3.

Supplementary Information 4.

Supplementary Information 5.

Supplementary Information 6.

Supplementary Information 7.

Supplementary Information 8.

Supplementary Information 9.

Supplementary Information 10.

Supplementary Information 11.

Supplementary Information 12.

Supplementary Information 13.

Supplementary Information 14.

## Data Availability

All data analyzed during this study are included in this published article and in supplementary files.
