# Supplementary material for: Distinct DNA methylation patterns associated with treatment resistance in metastatic castration resistant prostate cancer
Source: Sci Rep. 2021 Mar 23;11:6630. doi: 10.1038/s41598-021-85812-3 (PMC7988053; doi:10.1038/s41598-021-85812-3)
Supplement: Supplementary file 1 — Supplementary Information 1. [file 41598_2021_85812_MOESM1_ESM.pdf]

Distinct DNA methylation patterns associated with treatment resistance in metastatic castration resistant prostate cancer

Authors: Madonna R. Peter[1,2], Misha Bilenky[3], Alastair Davies[4], Ruth Isserlin[5], Gary D. Bader[5], Neil E. Fleshner[6], Martin Hirst[3,7], Amina Zoubeidi[4], Bharati Bapat[1,2]*

Affiliations:

1. Lunenfeld-Tanenbaum Research Institute, Sinai Health System, Toronto, Canada.
2. Department of Laboratory Medicine & Pathobiology, University of Toronto, Toronto, Canada.
3. Canada's Michael Smith Genome Science Center, BC Cancer Agency, Vancouver, Canada.
4. Vancouver Prostate Centre, Vancouver, British Columbia, Canada.
5. Terrence Donnelly Centre for Cellular and Biomolecular Research, University of Toronto, Toronto, Canada.
6. Departments of Surgery and Surgical Oncology, Division of Urology, University Health Network, Toronto, Canada.
7. Department of Microbiology and Immunology and Michael Smith Laboratories, University of British Columbia, Vancouver, Canada.

*Corresponding author: Dr. Bharati Bapat. 60 Murray Street, Toronto, ON, Canada, M5T 3L9. Telephone: 416-586-4800, Ext: 5175. Email: bapat@lunenfeld.ca

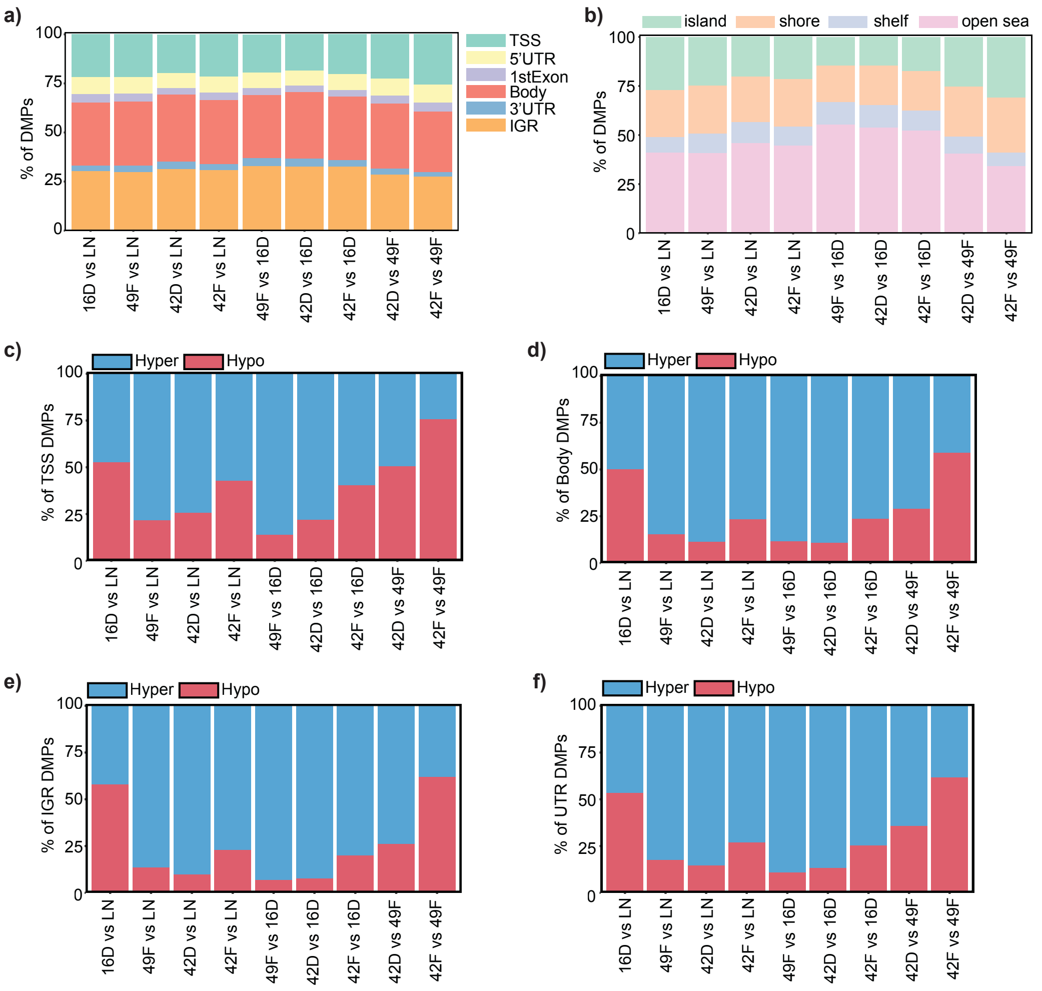

Supplementary Figure 1. Overview of DMPs in key genomic regions for all comparisons. (**a**) The distribution of DMPs for key genomic regions analyzed is shown, including transcription start sites (TSS), untranslated regions (UTR), gene bodies, and intergenic regions (IGR). (**b**) The proportion of DMPs within open sea regions, CpG islands (CGIs), CGI shores, and CGI shelves is shown. The distribution of methylation trends (hypermethylated and hypomethylated) for all comparisons is summarized for (**c**) TSS regions, (**d**) gene bodies, (**e**) intergenic regions, and (**f**) UTRs.

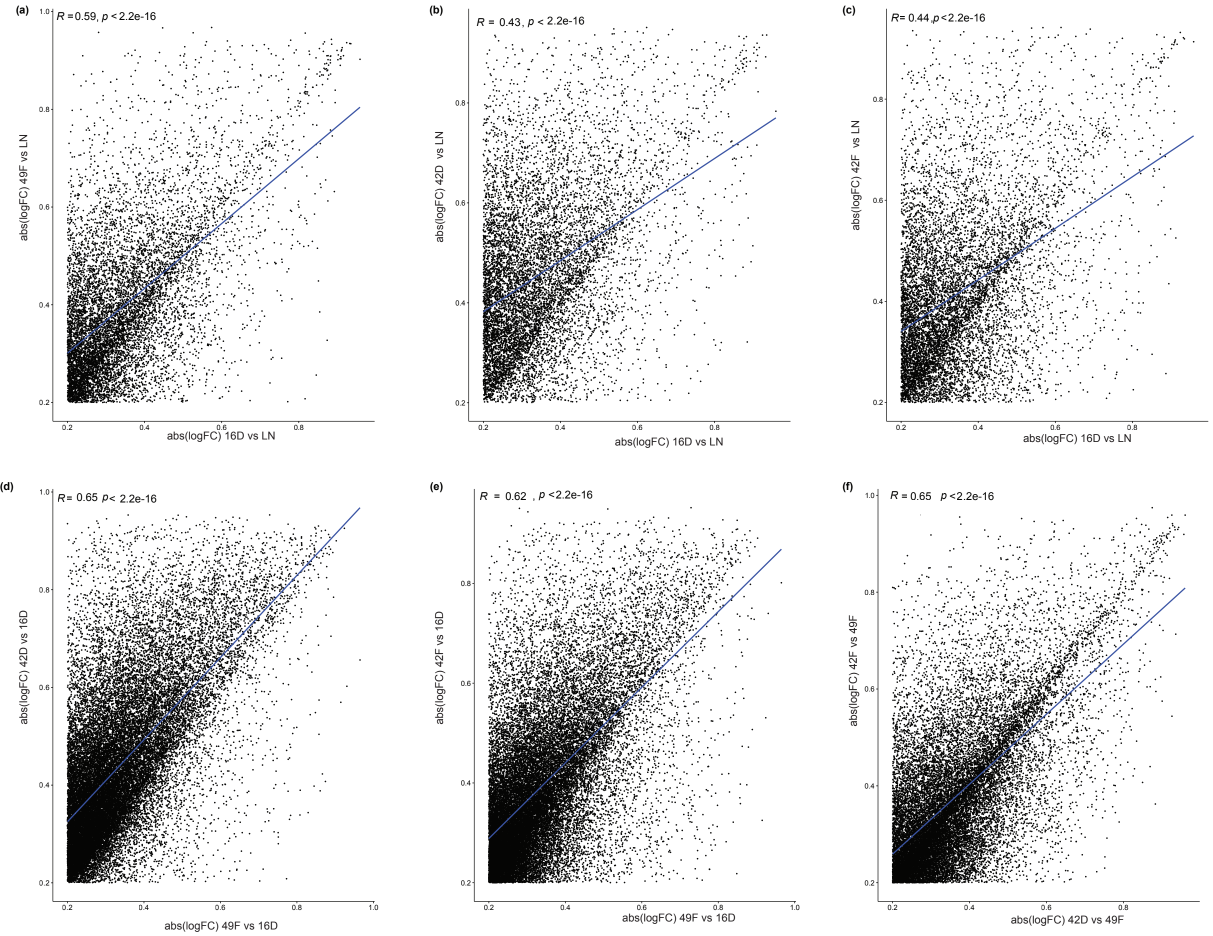

Supplementary Figure 2. Concordance analysis of commonly shared DMPs.

For all DMPs shared between (**a**) 49F^{ENZR} vs LN and 16D^{CRPC} vs LN, (**b**) 42D^{ENZR} vs LN and 16D^{CRPC} vs LN, (**c**) 42F^{ENZR} vs LN and 16D^{CRPC} vs LN, (**d**) 42D^{ENZR} vs 16D^{CRPC} and 49F^{ENZR} vs 16D^{CRPC}, (**e**) 42F^{ENZR} vs 16D^{CRPC} and 49F^{ENZR} vs 16D^{CRPC}, and (**f**) 42D^{ENZR} vs 49F^{ENZR} and 42F^{ENZR} vs 49F^{ENZR} is shown (Pearson correlation coefficients and p-values are shown for each plot).

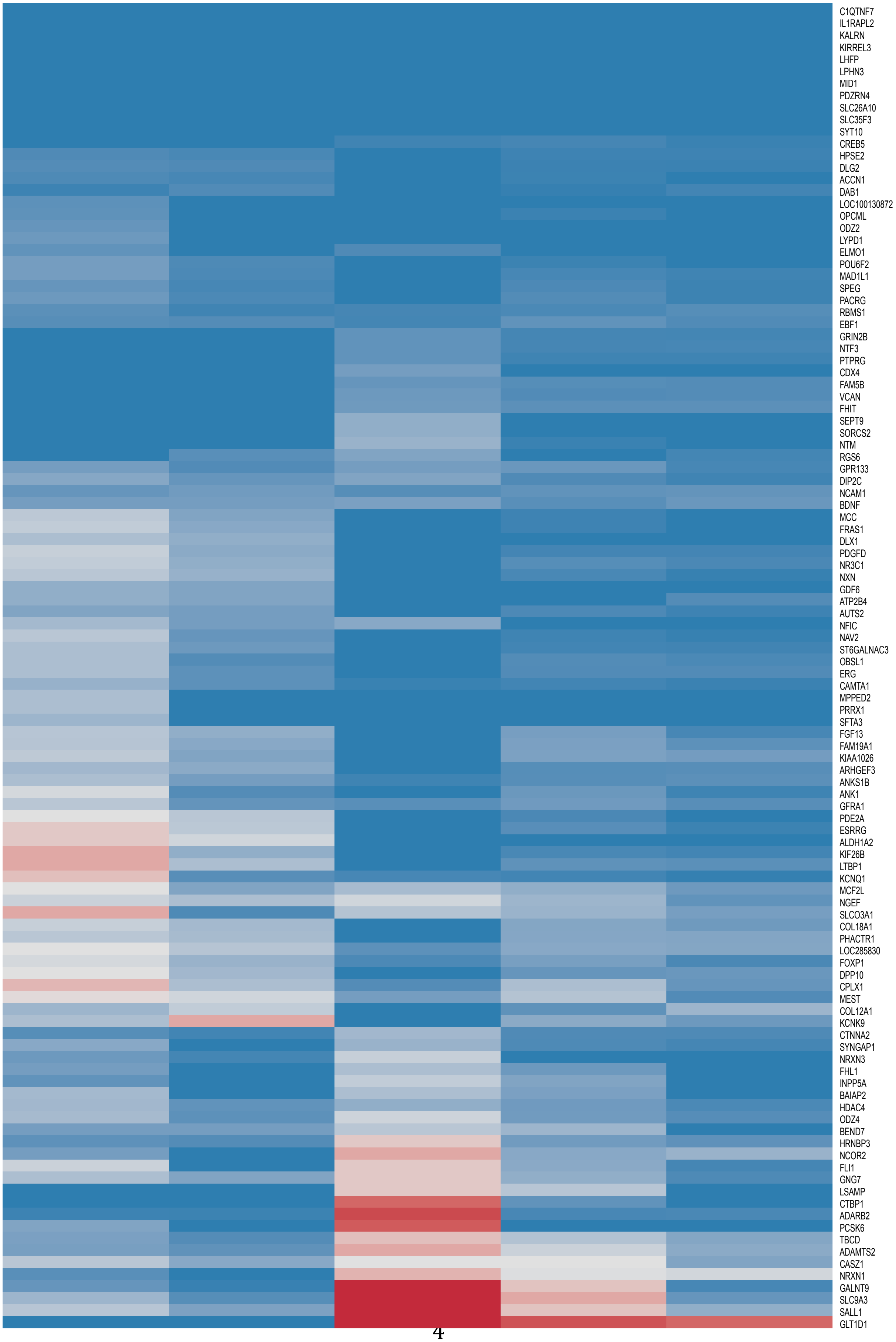
C1QTNF7
IL1RAPL2
KALRN
KIRREL3
LHFP
LPHN3
MID1
PDZRN4
SLC26A10
SLC35F3
SYT10
CREB5
HPSE2
DLG2
ACCN1
DAB1
LOC100130872
OPCML
ODZ2
LYPD1
ELMO1
POU6F2
MAD1L1
SPEG
PACRG
RBMS1
EBF1
GRIN2B
NTF3
PTPRG
CDX4
FAM5B
VCAN
FHIT
SEPT9
SORCS2
NTM
RGS6
GPR133
DIP2C
NCAM1
BDNF
MCC
FRAS1
DLX1
PDGFD
NR3C1
NXN
GDF6
ATP2B4
AUTS2
NFIC
NAV2
ST6GALNAC3
OBSL1
ERG
CAMTA1
MPPED2
PRRX1
SFTA3
FGF13
FAM19A1
KIAA1026
ARHGEF3
ANKS1B
ANK1
GFRA1
PDE2A
ESRRG
ALDH1A2
KIF26B
LTBP1
KCNQ1
MCF2L
NGEF
SLCO3A1
COL18A1
PHACTR1
LOC285830
FOXP1
DPP10
CPLX1
MEST
COL12A1
KCNK9
CTNNA2
SYNGAP1
NRXN3
FHL1
INPP5A
BAIAP2
HDAC4
ODZ4
BEND7
HRNBP3
NCOR2
FLI1
GNG7
LSAMP
CTBP1
ADARB2
PCSK6
TBCD
ADAMTS2
CASZ1
NRXN1
GALNT9
SLC9A3
SALL1
GLT1D1

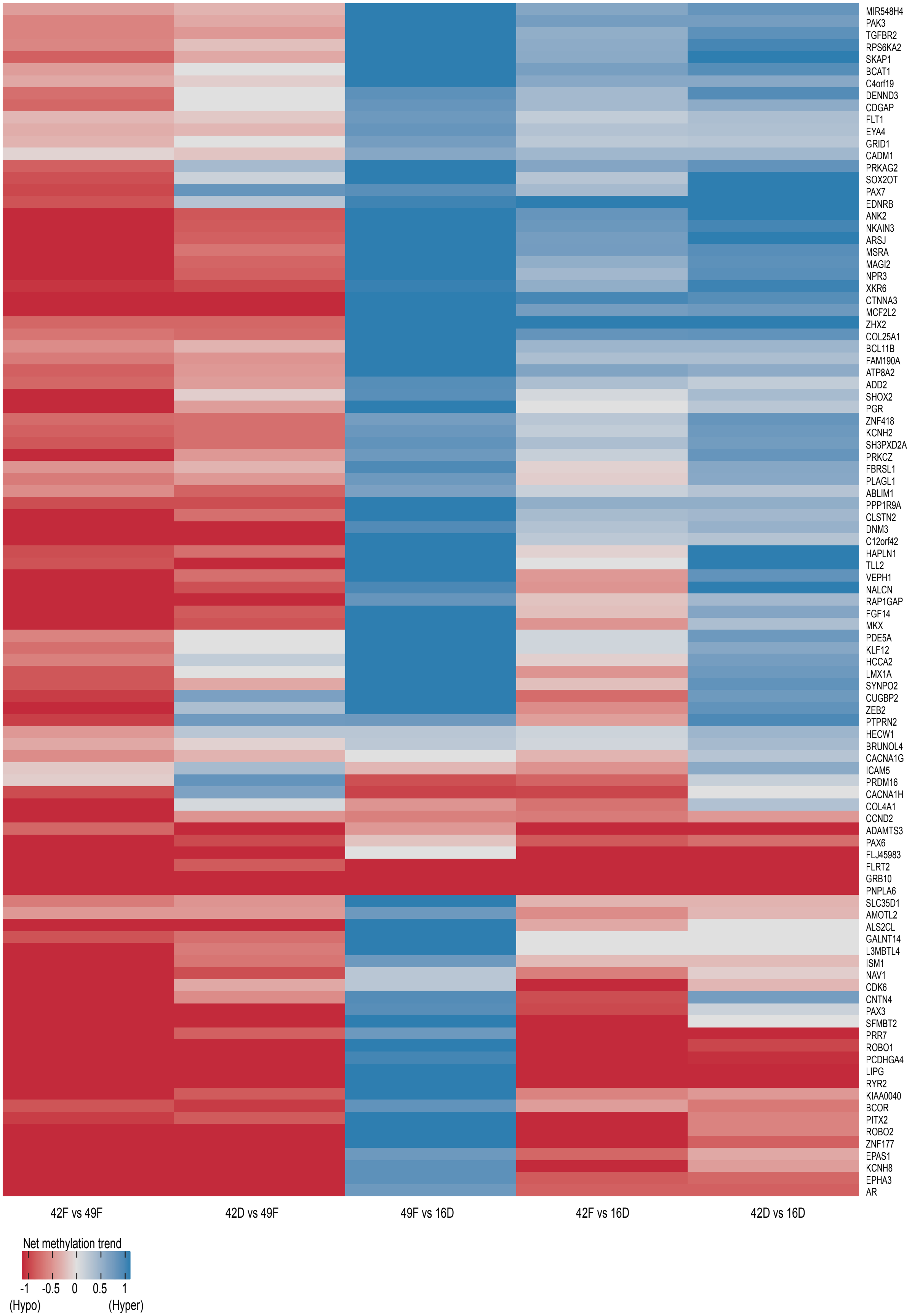
MIR548H4
PAK3
TGFBR2
RPS6KA2
SKAP1
BCAT1
C4orf19
DENND3
CDGAP
FLT1
EYA4
GRID1
CADM1
PRKAG2
SOX2OT
PAX7
EDNRB
ANK2
NKAIN3
ARSJ
MSRA
MAGI2
NPR3
XKR6
CTNNA3
MCF2L2
ZHX2
COL25A1
BCL11B
FAM190A
ATP8A2
ADD2
SHOX2
PGR
ZNF418
KCNH2
SH3PXD2A
PRKCZ
FBRSL1
PLAGL1
ABLIM1
PPP1R9A
CLSTN2
DNM3
C12orf42
HAPLN1
TLL2
VEPH1
NALCN
RAP1GAP
FGF14
MKX
PDE5A
KLF12
HCCA2
LMX1A
SYNPO2
CUGBP2
ZEB2
PTPRN2
HECW1
BRUNOL4
CACNA1G
ICAM5
PRDM16
CACNA1H
COL4A1
CCND2
ADAMTS3
PAX6
FLJ45983
FLRT2
GRB10
PNPLA6
SLC35D1
AMOTL2
ALS2CL
GALNT14
L3MBTL4
ISM1
NAV1
CDK6
CNTN4
PAX3
SFMBT2
PRR7
ROBO1
PCDHGA4
LIPG
RYR2
KIAA0040
BCOR
PITX2
ROBO2
ZNF177
EPAS1
KCNH8
EPHA3
AR
42F vs 49F
42D vs 49F
49F vs 16D
42F vs 16D
42D vs 16D
Net methylation trend
-1
-0.5
0
0.5
1
(Hypo)
(Hyper)

Supplementary Figure 3. Key genes with DMPs during the development of ENZR and tNEPC states.

Heatmap shows net methylation changes in genes central to all comparisons and with 5 or more DMPs. Genes with all/majority of DMPs hypermethylated were labeled blue and those with all/majority of DMPs in a hypomethylated state are shown in red.

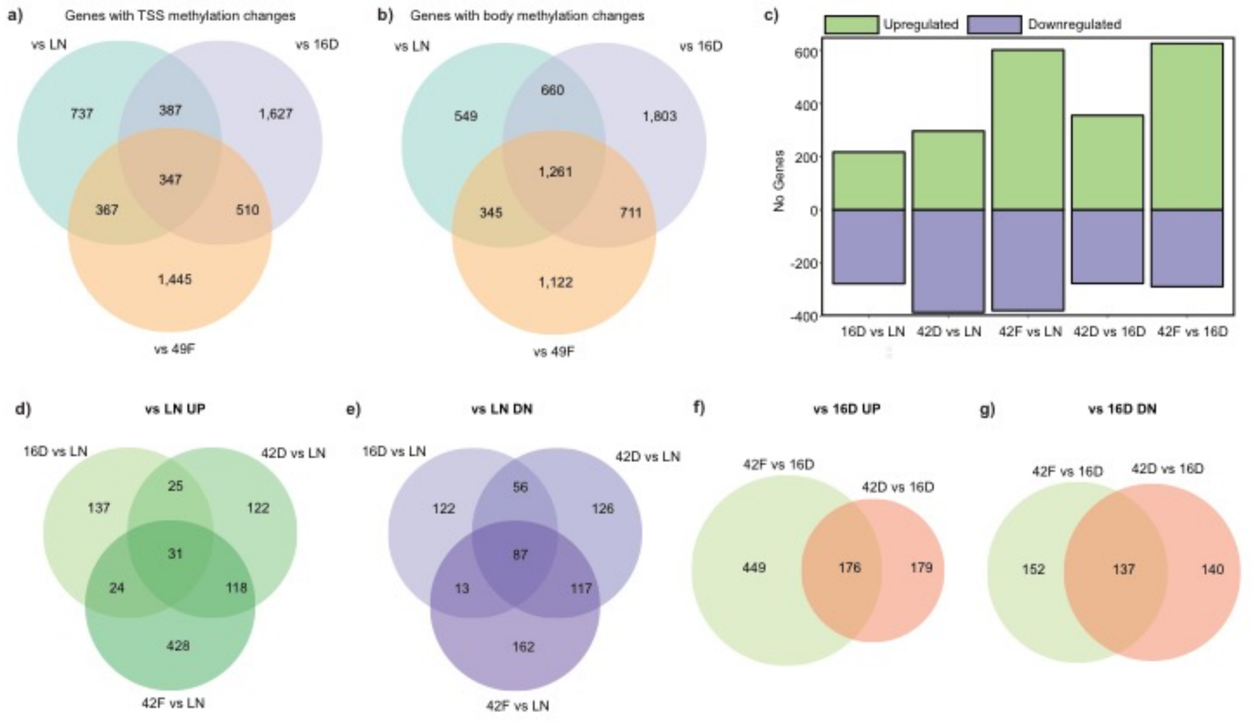

Supplementary Figure 4. Summary of common genes with DNA methylation changes and gene expression alterations.

(**a**) Venn diagram shows the number of genes with DMPs in TSS sites and the overlap between comparisons. (**b**) The extent of overlap of genes with DMPs in gene bodies between comparisons is also shown. (**c**) Overview of the number of genes that were either upregulated or downregulated in expression. Venn diagrams show the overlap of (**d**) upregulated or (**e**) downregulated genes across all vs LN comparisons. Similar distribution of genes (**f**) upregulated and (**g**) downregulated genes in tNEPC cells is shown.

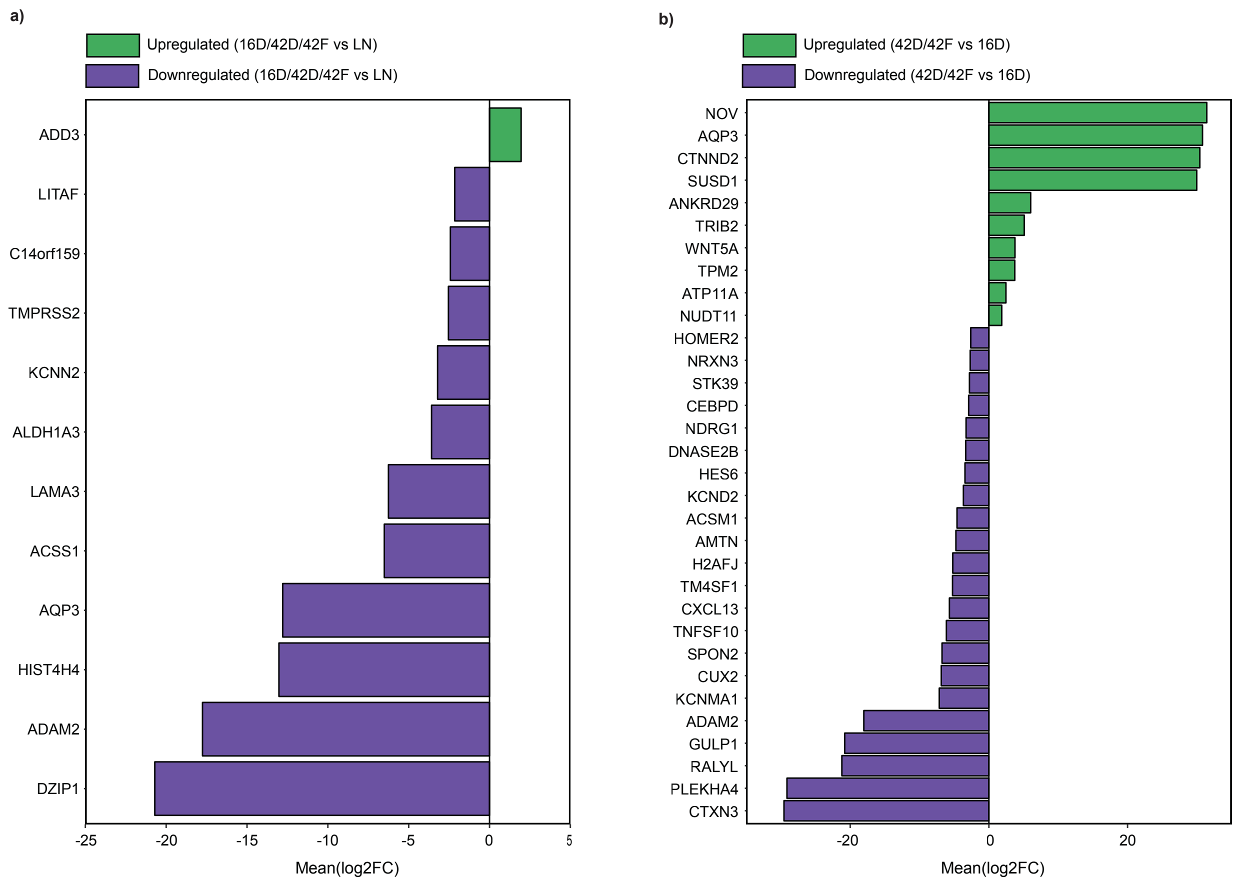

Supplementary Figure 5. Expression changes in androgen pathway related genes.

(**a**) Barplot shows the mean log2FC of AR associated genes commonly altered in all CRPC cell lines vs LN. (**b**) Similarly, differentially expressed AR-related genes in tNEPC cells vs 16D^{CRPC} cells are summarized.

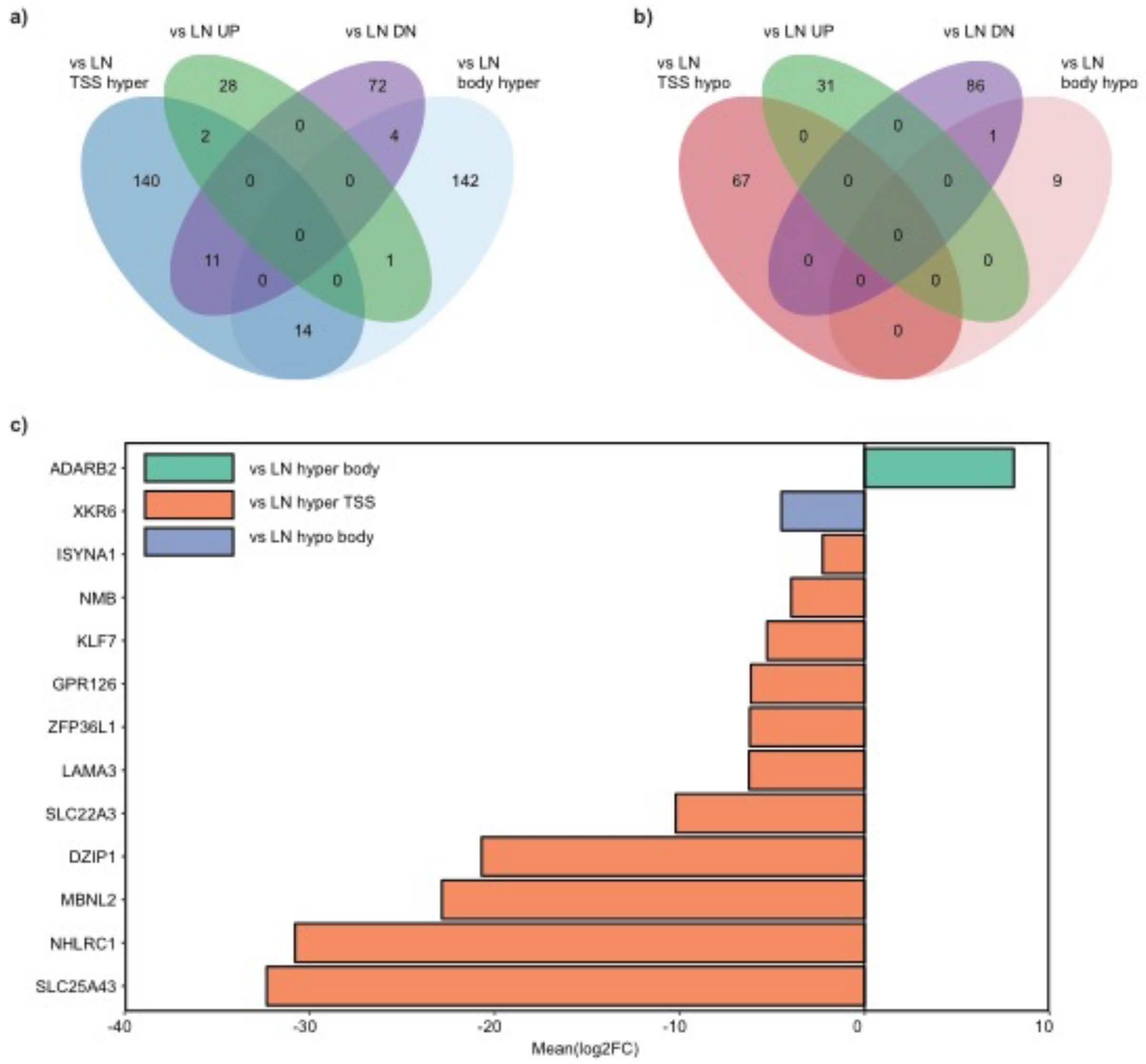

Supplementary Figure 6. Integrating common CRPC-related DNA methylation changes with differentially expressed genes.

(**a**) Venn diagram examines genes that hypermethylated in all vs LN TSS comparisons and/or gene bodies as well as with differential expression (UP = upregulated genes in $16D^{CRPC}/42D^{ENZR}/42F^{ENZR}$ vs LN, DN = downregulated genes). (**b**) Similarly, the extent of overlap between vs LN hypomethylated genes is shown. (**c**) Bar plot shows all genes with differential expression and associated changes in DNA methylation, including upregulated genes with hypermethylation in gene bodies in $16D^{CRPC}/42D^{ENZR}/42F^{ENZR}$ vs LN comparisons, those

hypomethylated in gene bodies with downregulated expression, as well as hypermethylated in TSS regions (not bodies) with decreased expression.

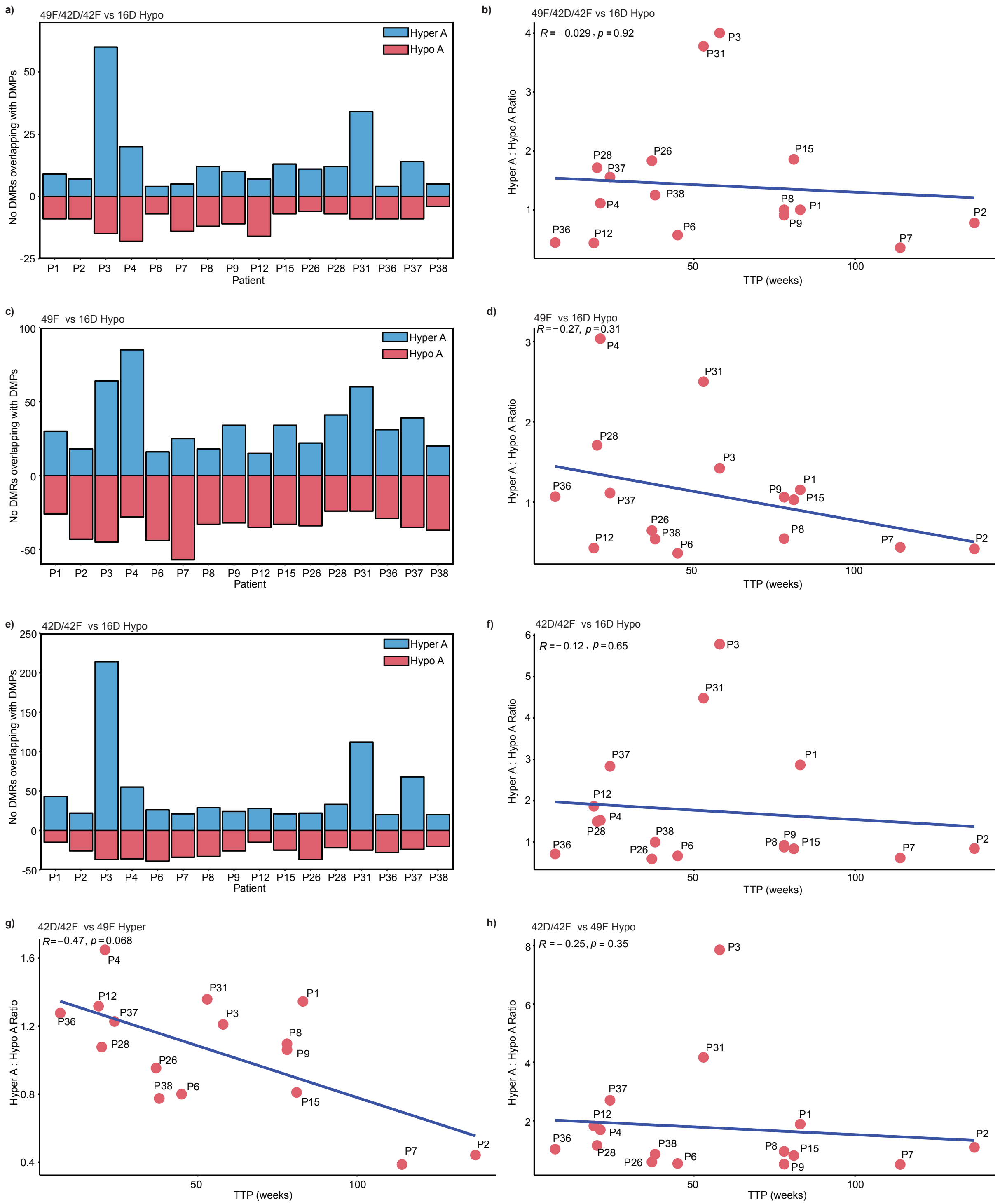

Supplementary Figure 7. Additional DMP comparisons with cfDNA DMRs.

The DMPs that overlapped with cfDNA DMRs obtained from comparing pre-treatment visits (visit A) and 12-week during treatment visits (Visit B) was assessed. (**a**) The number of DMRs

that overlapped with hypomethylated DMPs from comparing $49F^{ENZR}/42D^{ENZR}/42F^{ENZR}$ vs $16D^{CRPC}$ was quantified and separated by methylation trend (hypermethylated or hypomethylated in visit A vs B). (**b**) The ratio of these DMRS (hypermethylated to hypomethylated) was correlated with TTP (Spearman rho and p value is shown). (**c**) Similarly, the number of DMRs that overlapped with $49F^{ENZR}$ vs $16D^{CRPC}$ hypomethylated DMPs (not found in $42D^{ENZR}/42F^{ENZR}$) is shown in the bar plot, and (**d**) correlation analysis with TTP. (**e**) The number of DMRs that contained hypomethylated DMPs from $42D^{ENZR}/42F^{ENZR}$ vs $16D^{CRPC}$ (not $49F^{ENZR}$) was calculated, and (**f**) correlated with TTP. Additional Spearman correlations with TTP were also performed by comparing cfDNA DMRs with (**g**) $42D^{ENZR}/42F^{ENZR}$ vs $49F^{ENZR}$ hypermethylated DMPs and (**h**) $42D^{ENZR}/42F^{ENZR}$ vs $49F^{ENZR}$ hypomethylated DMPs.
